# The chromatin-modifying protein HUB2 is involved in the regulation of lignin composition in xylem vessels

**DOI:** 10.1093/jxb/eraa264

**Published:** 2020-06-01

**Authors:** Bo Zhang, Bernadette Sztojka, Carolin Seyfferth, Sacha Escamez, Pál Miskolczi, Maxime Chantreau, László Bakó, Nicolas Delhomme, András Gorzsás, Rishikesh P Bhalerao, Hannele Tuominen

**Affiliations:** 1 Umeå Plant Science Centre, Department of Plant Physiology, Umeå University, Umeå, Sweden; 2 Umeå Plant Science Centre, Department of Forest Genetics and Plant Physiology, Swedish University of Agricultural Sciences, Umeå, Sweden; 3 Department of Chemistry, Umeå University, Umeå, Sweden; 4 University of Manchester, UK

**Keywords:** Arabidopsis, cell wall chemistry, HUB2, lignin, PIRIN2, syringyl-type lignin, xylem vessels

## Abstract

PIRIN2 (PRN2) was earlier reported to suppress syringyl (S)-type lignin accumulation of xylem vessels of *Arabidopsis thaliana*. In the present study, we report yeast two-hybrid results supporting the interaction of PRN2 with HISTONE MONOUBIQUITINATION2 (HUB2) in Arabidopsis. HUB2 has been previously implicated in several plant developmental processes, but not in lignification. Interaction between PRN2 and HUB2 was verified by β-galactosidase enzymatic and co-immunoprecipitation assays. HUB2 promoted the deposition of S-type lignin in the secondary cell walls of both stem and hypocotyl tissues, as analysed by pyrolysis-GC/MS. Chemical fingerprinting of individual xylem vessel cell walls by Raman and Fourier transform infrared microspectroscopy supported the function of HUB2 in lignin deposition. These results, together with a genetic analysis of the *hub2 prn2* double mutant, support the antagonistic function of PRN2 and HUB2 in deposition of S-type lignin. Transcriptome analyses indicated the opposite regulation of the S-type lignin biosynthetic gene *FERULATE-5-HYDROXYLASE1* by PRN2 and HUB2 as the underlying mechanism. PRN2 and HUB2 promoter activities co-localized in cells neighbouring the xylem vessel elements, suggesting that the S-type lignin-promoting function of HUB2 is antagonized by PRN2 for the benefit of the guaiacyl (G)-type lignin enrichment of the neighbouring xylem vessel elements.

## Introduction

Lignin is a polyphenolic polymer which is built up from mostly three different types of monolignols: guaiacyl (G), syringyl (S), and *p*-hydroxyphenyl (H) units (reviewed by Boerjan *et al.*, 2003). Along with cellulose and hemicelluloses, lignin comprises a major part of the plant secondary cell walls. During development, lignin is deposited in the walls of a variety of cell types, such as tracheary elements, sclerenchyma cells, endodermal cells, seed coat cells, and silique valve margin cells (Barros *et al.*, 2015). In addition to the developmental programmes, lignification is triggered by different biotic and abiotic stress factors such as wounding, pathogen attack, temperature, metabolic stress, and perturbation of the cell wall structure (Cano-Delgado *et al*., 2003; Tronchet *et al.*, 2010). Monolignols are synthesized through the phenylpropanoid pathway, starting with phenylalanine that is deaminated, followed by hydroxylation and methoxylation reactions of the aromatic ring, and conversion of a carboxylic acid moiety to an alcohol (Boerjan *et al.*, 2003).

The transcriptional regulation of lignin biosynthetic genes is under the control of several different types of transcription factors, including the MYB and NAC family transcription factors (for recent reviews, see Nakano *et al.*, 2015; Ohtani and Demura, 2019). Many of these transcription factors operate in regulatory networks that are multilayered and highly branched (Hussey *et al.*, 2013; Taylor-Teeples *et al.*, 2015). MYB46 and MYB83 were shown to bind to the secondary wall MYB-responsive element (SMRE) motifs, and thus directly control an array of not only other transcription factors controlling secondary cell wall formation and lignification (MYB58 and MYB63), but also lignin biosynthetic genes themselves (Zhong and Ye, 2012). MYB58 and MYB63 transcriptionally activate lignin biosynthetic genes, as well as the lignin-polymerizing enzyme LACCASE4, through binding to the AC *cis*-regulatory elements of the promoters. *CINNAMATE-4-HYDROXYLASE* (*C4H*) and *CAFFEIC ACID 3-Ο-METHYLTRANSFERASE* (*COMT*), two monolignol biosynthetic genes, were also directly activated by MYB58 and MYB63, even though their promoters may only contain deteriorated AC elements. Similar AC element-dependent activation of lignin biosynthetic genes was shown for MYB85 (Zhou *et al.*, 2009). The transcriptional regulation of *FERULATE 5-HYDROXYLASE* (*F5H*), an evolutionary recent addition to the monolignol biosynthetic pathway generating S-type lignin precursors (Zhao *et al.*, 2010), differs from the other lignin biosynthetic genes. The *F5H* promoter does not contain an apparent AC *cis*-element, and it is not believed to be regulated by MYB58 and MYB63 (Zhou *et al.*, 2009). Instead,Öhman *et al*. (2013) showed that the expression of *F5H* and thus S-type lignin biosynthesis is regulated, possibly indirectly, by MYB103. Furthermore, in *Medicago truncatula*, the NAC transcription factor MtNST1 regulates *F5H* expression by direct binding to its promoter (Zhao *et al.*, 2010).

The function of transcription factors is influenced by the chromatin status of the transcription factor-binding sites. Different chromatin modifications are frequently linked to transcriptional activity of genes regulating specific developmental processes. Surprisingly little is known about the chromatin level regulation of lignification. In Eucalyptus, the function of the MYB transcription factor EgMYB1 in repressing lignin biosynthetic genes is enhanced by binding to the linker histone variant H1.3 (Soler *et al*., 2017). An association between the level of transcriptionally activating (H3K4me3) and repressive (H3K27me3) marks and the expression level of some lignin biosynthetic genes was demonstrated in the xylem tissues of Eucalyptus trees (Hussey *et al*., 2015, 2017). During early xylem development, lignin and phenylpropanoid biosynthetic genes were enriched with the repressive H3K27me3 marks, in agreement with the lack of lignin deposition at this stage (Hussey *et al.*, 2017). Chromatin level regulation was also shown for BLUE-COPPER-BINDING PROTEIN (BCB) which affects accumulation of lignin and plant freezing tolerance in Arabidopsis (Ji *et al.*, 2015).

We recently showed that the PIRIN2 (PRN2) protein suppresses S-type lignin biosynthesis in the secondary cell walls of xylem vessels in Arabidopsis (Zhang *et al.*, 2020*a*). PIRIN2 belongs to a rather poorly described cupin domain-containing family of proteins with four members in Arabidopsis (Zhang *et al.*, 2020*a*). Our data supported that PRN2 regulates lignification by controlling the transcription of lignin biosynthetic genes. To corroborate this and to further elucidate the molecular network of PRN2, we searched for protein interactors of PRN2. We present here the interactors of PRN2, and characterize one of them, HISTONE MONOUBIQUITINATION2 (HUB2), in detail in connection with lignification. In Arabidopsis, HUB2 and HUB1 are E3 ubiquitin ligases which together with the E2 enzymes UBIQUITIN CARRIER PROTEIN1 (UBC1) and UBC2, catalyse histone H2B monoubiquitination (H2Bub1) of chromatin (Fleury *et al.*, 2007; Liu *et al.*, 2007; Cao *et al.*, 2008). HUB2 was previously implicated in several aspects of plant development, such as flowering time, seed dormancy, circadian clock (Fleury *et al.*, 2007; Liu *et al.*, 2007; Cao *et al.*, 2008; Xu *et al.*, 2009; Himanen *et al.*, 2012; Gu *et al*., 2013; Zhao *et al.*, 2019), leaf cuticle formation (Ménard *et al.*, 2014), drought tolerance (Chen *et al.*, 2019), anther development (Cao *et al.*, 2015), defence response, and pathogen resistance (Zou *et al.*, 2014; Zhang *et al*., 2015; Zhao *et al.*, 2020). In this study, we reveal the involvement of HUB2 in the regulation of lignification in Arabidopsis (*Arabidopsis thaliana*). Transcriptomic analysis indicates that HUB2 promotes S-type lignin biosynthesis by influencing the expression of *F5H* in the secondary xylem tissues.

## Materials and methods

### Plant material and growth conditions

Arabidopsis T-DNA insertion mutants used in this study included *prn2-1* (SM_3.15394), *prn2-2* (SALK_079571), *hub2-1* (GABI_634H04), *hub2-2* (SALK_071289), *c4h-3* (ref3.3), and *ccr1-3* (SALK_123689), which all have been described previously (Ruegger and Chapple, 2001; Fleury *et al.*, 2007; Cao *et al.*, 2008;Mir Derikvand *et al*., 2008; Zhang *et al.*, 2020*a*). The *PRN2*-overexpressing lines 6 and 13 (overexpression under the control of the 35S promoter) have been described in Zhang *et al*. (2014, 2020*a*).

Plants were grown on soil in growth chambers under short-day conditions (8 h light/16 h dark, 21 °C/18 °C, 70% relative humidity) for 4–8 weeks and then moved to long-day conditions (16 h light/8 h dark, 21 °C/18 °C, 70% relative humidity). When the inflorescence stems reached a height of 50 cm, the 2 cm bottom part of the stem and the hypocotyl were harvested for the different analyses.

### Yeast two-hybrid analysis

Yeast two-hybrid screening was performed by Hybrigenics S.A., Paris, France, using the full-length coding sequence (CDS) of *PRN2* (At2g43120) as a bait. Targeted yeast two-hybrid interaction assays were performed as described previously (Zhang *et al.*, 2014).

### Transient expression in Arabidopsis root cell protoplasts and co-immunoprecipitation assay

Full-length CDS of *PRN2* and *HUB2* were cloned into the pRT104 3×HA or pRT104 3×myc vectors (Fülöp *et al.*, 2005) using *Bam*HI, *Eco*RI, and *Cla*I restriction enzymes. Transfection of Arabidopsis protoplasts and co-immunoprecipitation were performed as previously described (Meskiene *et al.*, 2003; Fülöp *et al.*, 2005). Expression of both constructs was allowed overnight. The myc-tagged proteins were immunoprecipitated from total protein extracts by incubating the extracts for 2 h at 4 °C with 500 ng of 9E10C anti-myc monoclonal antibody (Covance) and 10 μl of protein G–Sepharose (GE Healthcare). Beads were then washed three times with washing buffer [1× phosphate-buffered saline (PBS) pH 7.4, 5% glycerol, 0.1% Igepal CA-630] and bound proteins were eluted with 50 μl of SDS loading buffer. Co-immunoprecipitation of the HA-tagged protein was detected by SDS–PAGE followed by western blotting using the 16B12 anti-HA-POD (1:1000) monoclonal antibody (Roche) and enhanced chemiluminescence detection (SuperSignal WestPico, Pierce).

### Histochemical GUS staining

Transverse sections of tissue samples obtained by hand-sectioning from soil-grown Arabidopsis were used for histochemical β-glucuronidase (GUS) staining. Samples were fixed in 90% ice-cold acetone for 30 min, then incubated at 37 °C in the GUS solution [1 mM X-Gluc, 1 mM K_3_Fe(CN)_6_, 1 mM KR_4_Fe(CN)_6_, and 0.1% Triton X-100 in 50 mM sodium phosphate buffer (Na_2_HPO_4_/NaH_2_PO_4_, pH 7.2)]. The samples were destained in a 99% ethanol solution and gradually rehydrated with a wash series of decreasing ethanol concentration. A Zeiss Axioplan II light microscope equipped with an AxioCam CCD camera (Zeiss, Jena, Germany) was used for image acquisition.

### Pyrolysis–GC/MS (Py-GC/MS)

Pools of freeze-dried Arabidopsis stem and hypocotyl samples were ball-milled as described by Zhang *et al.* (2020*a*). A 60±10 μg aliquot of ball-milled powder was then applied to the pyrolizer (PY-2020iD and AS1020E, FrontierLabs, Japan) attached to a GC/MS (7890A/5975C, Agilent Technologies AB Sweden, Kista, Sweden). The pyrolysate was separated, and the data processing and analysis were performed according to Gerber *et al.* (2012). Each sample pool (called here biological replicates) consisted of three stem pieces (the 2 cm bottom part) or hypocotyls, and a minimum of three biological replicates per genotype were always analysed.

### Transmission electron microscopy

Sections for TEM were prepared as described in Bollhöner *et al.* (2013), and imaged with a Jeol 1230 transmission electron microscope and Gatan MSC 600CW camera.

### FT-IR and Raman microspectroscopy

Transverse sections (20 μm thick) were cryo-sectioned from the hypocotyls of 8-week-old Arabidopsis plants. Fourier transfer infared (FT*-*IR) and Raman microspectroscopic measurements were performed according to previous protocols (Gorzsás *et al*., 2011, 2017) and experimental parameters (Zhang *et al.*, 2020*a*). For FT-IR spectra, section thickness resulted in saturation in the carbohydrate region, limiting the spectral range used in the analysis (see below). Recorded spectra were exported to Matlab (v. 17a-18b, Matworks, CA, USA) for processing (baseline correction, total area normalization, and Savitzky–Golay smoothing) using the free, open-source script developed at the Vibrational Spectroscopy Core Facility at Umeå University (https://www.umu.se/en/research/infrastructure/visp/downloads/), before being imported to SIMCA-P (v. 16, Sartorius Stedim Data Analytics AB, Sweden) for OPLS-DA (orthogonal projections to latent structures discriminant analysis). The following processing parameters were used for FT-IR/Raman spectra: spectral range=1180–1800 cm^−1^/630–1900 cm^−1^; asymmetric least squares baseline lambda=100 000 and *P*=0.001 for both; Savitzky–Golay smoothing with first-order polynomial and a frame of five for both. Data were centred in SIMCA-P and the entire spectral region was used for the analyses, with a fixed number of components (one predictive and two orthogonal) in all pairwise comparisons.

### ChIP-qPCR

The 2 cm bottom part of stems from 8-week-old plants were harvested and immediately cross-linked for 20 min in 40 ml of 1% formaldehyde buffer under vacuum at room temperature. Fifteen plants were pooled as one biological replicate, and three biological replicates for each line were used for the assays. The chromatin was isolated as previously described (Saleh *et al.*, 2008) and sheared by using a Bioruptor UCD-300 sonicator (Diagenode) to reduce the average DNA length to 500 bp. The sonicated chromatin was diluted 10 times by chip dilution buffer, immunoprecipitated by 5 μg of antibody bound to Dynabeads Protein A or Protein G (Invitrogen), and incubated overnight at 4 °C. Anti-histone H2Bub1 (MM-0029-P, Medimabs) or rabbit IgG (ab37415, Abcam) as a control were used for immunoprecipitation. The beads were washed with low salt buffer (20 mM Tris–HCl at pH 8.0, 2 mM EDTA, 150 mM NaCl, 1% Triton X-100, 0.1% SDS) and high salt buffer (20 mM Tris–HCl at pH 8.0, 2 mM EDTA, 500 mM NaCl, 1% Triton X-100, 0.1% SDS), followed by LiCl buffer (10 mM Tris–HCl at pH 8.0, 1 mM EDTA, 0.25 M LiCl, 1% Igepal CA-630, 1% deoxycholate) and TE buffer (10 mM Tris–HCl at pH 8.0, 1 mM EDTA), and eluted with elution buffer (1% SDS and 0.1 M NaHCO_3_). The eluates were reverse cross-linked and treated with proteinase K (Fermentas) for 2 h at 55 °C and phenol/chloroform extracted. The DNA was recovered by ethanol precipitation, dissolved in water, and analysed by quantitative PCR (qPCR) using the LightCycler 480 SYBR Green I Master (Roche) reagent on a CFX96 real-time system (Bio-Rad). The genomic fragments analysed by qPCR were at the following distance from the start codon: *PAL1* 1945–2149 bp, *C4H* 1327–1432 bp, *HCT* 1194–1294 bp, *COMT* 78–230 bp, and *F5H* 716–947 bp. Primer sequences are listed in Supplementary Table S2 at *JXB* online.

### RNA isolation

Total RNA was isolated from pools of five hypocotyls using an RNeasy plant mini kit (Qiagen) according to the manufacturer’s protocol. All samples were treated by the DNA-free™ Kit (Invitrogen) to remove any remaining DNA. The RNA purity was checked by NanoDrop™ 2000 (ThermoScientific), the RNA quantity was determined by the Qubit 2.0 fluorometer (Invitrogen), and the integrity of the RNA was evaluated by the Agilent 2100 Bioanalyzer with Agilent RNA 6000 Nano Chips according to the manufacturer’s instructions.

### RNA-seq data analysis

Library preparation and paired-end Illumina sequencing was performed by Novogene (China). Raw data can be downloaded from the European Nucleotide Archive (ENA, U https://www.ebi.ac.uk/ena) under the accession PRJEB35753. RNA reads were filtered by removing rRNAs and sequencing adaptors (SortMeRNA, Kopylova *et al.*, 2012), followed by read trimming (Trimmomatic, Bolger *et al.*, 2014). The remaining reads were aligned to the latest *A. thaliana* genome version (ARAPORT11) using STAR (Dobin *et al.*, 2013). Reads were mapped using Kallisto (Bray *et al.*, 2016) and afterwards used to generate the counts per gene per library matrix using the R (v3.5.3) package EdgeR (v3.24.3). Reads with <10 counts in at least one library were discarded from the data set prior to count normalization (using the function calcNormFactors), library size correction, and log/transformation (using the function voom). Gene expression values [log_2_] in all four genotypes were calculated using a linear model with genotype as fixed effect and replicate as random effect [using the lmfit function from limma (v.3.38.3)], and variance shrinkage was applied. Differentially regulated genes in each mutant compared with the wild type (WT) were selected using a q-value cut-off of 0.05 and a log_2_fold change of < –1 and >1. Heatmaps were generated using the gplots package (v3.0.1.1). Gene Ontology (GO) enrichment analysis was performed with the enrichment tool at http://atgenie.org/ (Sundell *et al.*, 2015).

All scripts used in this study are available under: https://github.com/CarSeyff/AtHUB2.git. The transcriptomic results are available in Dataset 2 at the Dryad Digital Repository (https://dx.doi.org/10.5061/dryad.mgqnk98wf).

### Statistical analyses

Welch’s *t*-tests were applied for the comparison of different genotypes with the WT, always assuming unequal variance. Protected post-ANOVA Fisher LSD tests (α=0.05) were performed for multiple pairwise comparisons of all lines/treatments within the same experiment.

### Accession numbers

Nucleotide sequence data from this article can be found in the Arabidopsis Genome Initiative under the following accession numbers: *PRN2* (*At2g43120*), *HUB2* (*At1g55250*), *PAL1* (*At2g37040*), *C4H* (*At2g30490*), *HCT* (*At5g48930*), *F5H1* (*At4g36220*), *COMT1* (*At5g54160*), *CCR1* (*At1g15950*), *RD21* (*At1g47128*), *XCP2* (*At1g20850*), *At2g05920*, *At4g24620*, *ITN* (*At3g12360*), *At1g63770*, *At2g25740*, *At3g07340*, *At1g60070*, *At5g23110*, *At5g07400*, *At1g56000*, *At5g07740*, *At1g42430*, and *At5g22450*.

## Results

### PRN2 interacts with HISTONE MONOUBIQUITINATION2 (HUB2) *in vitro* and *in vivo*

To investigate the molecular network of PRN2 in lignification, we performed a large-scale yeast two-hybrid screen using the full-length Arabidopsis PRN2 as a bait. The principal isolates included clones encoding 18 proteins (Dataset 1 available at Dryad).

**Fig. 1.**
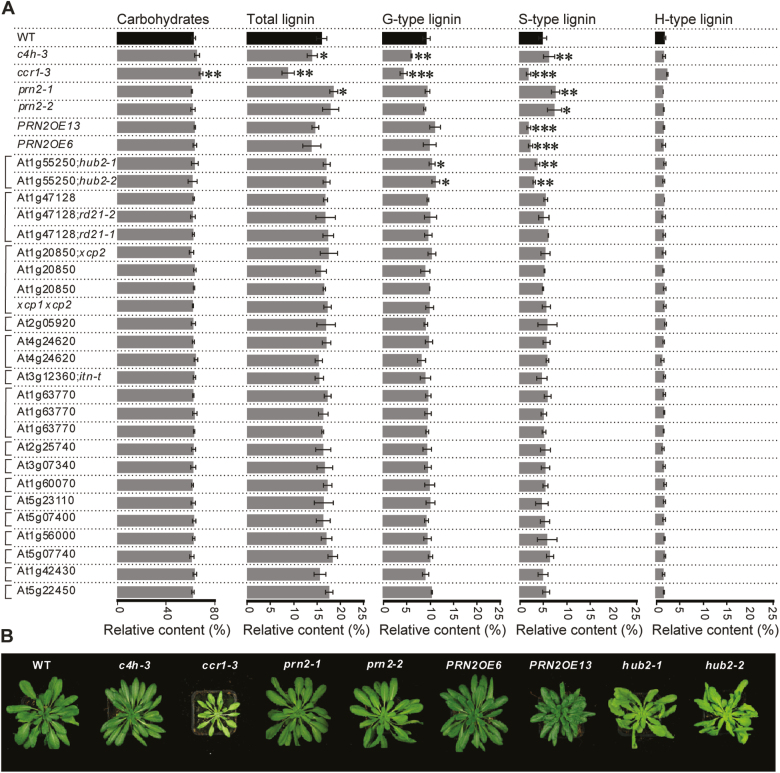
Mutants for one of the 16 PRN2 candidate interactors, HUB2, display a lignin phenotype. (A) Py-GC/MS analysis of the secondary cell wall composition in Arabidopsis hypocotyls. Relative content (%) of total lignin, G-type lignin, S-type lignin, and H-type lignin is based on their respective MS peak area as a proportion of the cumulative area from all peaks (Gerber *et al.*, 2012). Hypocotyl material was collected from 8-week-old soil-grown *prn2-1*, *prn2-2*, *PRN2*-overexpressor lines *PRN2OE6* and *PRN2OE13*, and the mutants corresponding to the potential interactors of PRN2. Two lignin monomer biosynthetic mutants, *ccr1-3* and *c4h-3*, were included as controls. For each genotype, five biological replicates were analysed, each composed of a pool of three hypocotyls. The asterisks indicate a statistically significant difference from the WT by Welch’s *t*-test (*P<0.05, **P<0.01, ***P<0.001). Error bars indicate ±SD. (B) Representative photographs of WT, *c4h-3*, *ccr1-3*, *prn2-1*, *prn2-2*, *PRN2OE6*, *PRN2OE13*, *hub2-1*, and *hub2-2* plants after 6 weeks of growth under short-day conditions.

Mature hypocotyls of T-DNA insertion mutants for 16 candidate genes (Supplementary Table S1) were analysed together with *prn2* mutants and *PRN2* overexpressor lines by Py-GC/MS (Fig. 1A). The two *prn2* mutants showed increased S-lignin accumulation and the two *PRN2* overexpressor lines showed decreased S-lignin accumulation, in line with our earlier work (Zhang *et al.*, 2020*a*). Interestingly, from the 25 insertion lines that were examined, two lines, GK_634H04 and SALK_071289, showed a significantly decreased level of S-type lignin as well as an increased level of G-type lignin compared with the WT (Fig. 1A). Both of these lines are mutants in *HUB2*, previously named *hub2-1* and *hub2-2* (Fleury *et al.*, 2007; Cao *et al.*, 2008). These results support a function for HUB2 in S-type lignification in a manner that is opposite to PRN2 function. Notably, even though the *hub2* mutants had pale leaves (Fig. 1B), their secondary xylem anatomy and cell morphology were indistinguishable from those of the WT and *prn2* (Supplementary Fig. S1).

**Fig. 2.**
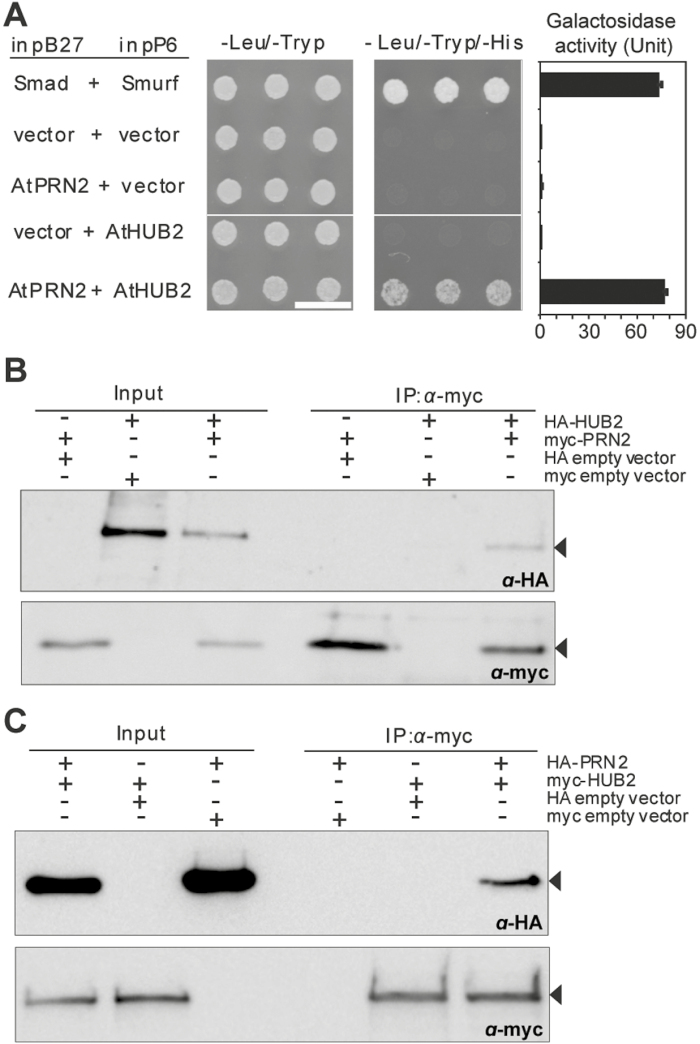
PRN2 interacts with HUB2. (A) Visualization of protein–protein interactions by yeast two-hybrid assay. The interaction of PRN2 with HUB2 was assessed by growth on plates with yeast growth medium lacking leucine, tryptophan, and histidine (-Leu/-Trp/-His). The two human proteins Smad+Smurf [encoding fusion protein LexA–Smad (pB27-Smad) and GAL4AD–Smurf (pP6-Smurf), respectively] were used as a positive control. The empty vectors pB27+pP6 vector, pB27-PRN2+empty pP6, and empty pB27+pP6-HUB2 were used as negative controls. Scale bar=1 cm. Activation of a second reporter gene (β-galactosidase) is shown to the right with a column chart. (B, C) Co-immunoprecipitation assays (co-IP) with anti-myc antibodies and detection by western blotting using anti-HA. Anti-myc monoclonal antibody co-immunoprecipitated HA-tagged HUB2 protein along with myc-PRN2 (B) and HA-tagged PRN2 along with myc-HUB2 (C). Inputs represent concentrated proteins in crude extracts. The labelled bands indicated by arrowheads refer to the co-precipitated HA-HUB2 (B) or HA-PRN2 (C).

Two independent approaches were used to further examine the interaction between PRN2 and HUB2 (Fig. 2). First, growth assays on a histidine dropout medium as well as enzymatic assays using the β-galactosidase (β-gal) reporter gene constructs revealed clear interaction between yeast strains harbouring the *PRN2* bait vector and the *HUB2* prey vector (Fig. 2A). Secondly, co-immunoprecipitation assays in Arabidopsis protoplasts provided *in vivo* support for the interaction. Using anti-myc monoclonal antibody, HA-tagged HUB2 co-immunoprecipitated with myc-tagged PRN2 (Fig. 2B) and, reciprocally, HA-tagged PRN2 co-immunoprecipitated with myc-tagged HUB2 (Fig. 2C). Thus, the assays confirmed the interaction between PRN2 and HUB2 both *in vitro* and *in vivo*.

### HUB2 and PRN2 interact genetically in S-lignin accumulation in hypocotyls

**Fig. 3.**
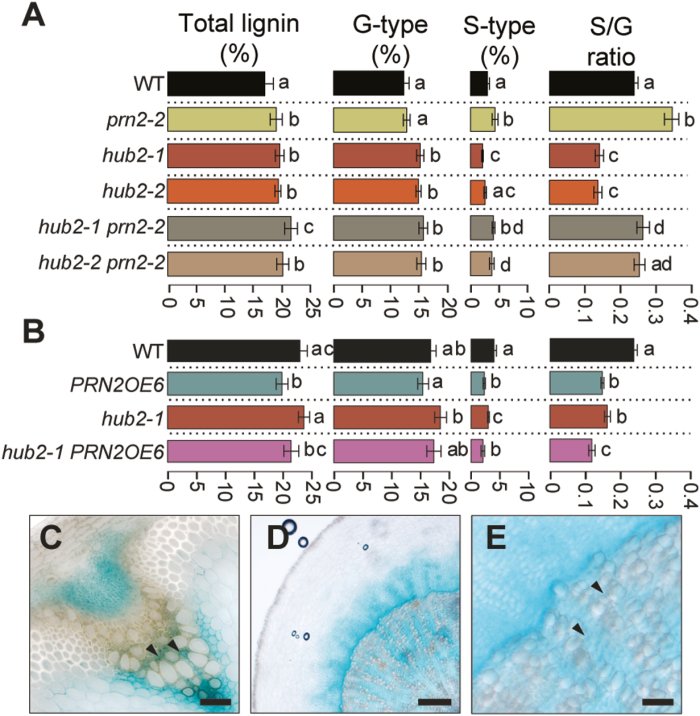
HUB2 affects the lignin content and composition of Arabidopsis hypocotyls. (A, B) The effect of HUB2 and PRN2 on the secondary cell wall composition in Arabidopsis hypocotyls. Py-GC/MS analysis was performed on hypocotyl material collected from 8-week-old *prn2-2*, *hub2-1*, *hub2-2*, *hub2-1 prn2-2*, *hub2-2 prn2-2*, *PRN2OE6*, and *hub2-1 PRN2OE6*. Relative contents (%) of total lignin, G-type lignin, and S-type lignin are based on their respective MS peak area as a proportion of the cumulative area from all peaks (Gerber *et al.*, 2012). For each genotype, five biological replicates were analysed, each composed of a pool of three hypocotyls. Lines that do not share any letter are significantly different from each other according to post-ANOVA Fisher’s test (*P*<0.05). Error bars indicate ±SD. (C–E) *HUB2* promoter activity. Histochemical β-glucuronidase (GUS) assay was performed on xylem tissues of plants expressing GUS under the control of the *HUB2* promoter. (C) Transverse section showing a vascular bundle in an inflorescence stem of a 6-week-old plant. (D, E) Transverse section from the hypocotyl of an 8-week-old plant. (E) Is a magnified image from (D). Arrowheads indicate xylem cells adjacent to lignifying vessel elements. The scale bar represents 100 µm (C, E) or 500 µm (D).

Genetic interaction between PRN2 and HUB2 was investigated by Py-GC/MS analysis of two *hub2* mutants, *prn2-2*, and *hub2 prn2* double mutants. The analyses of the hypocotyls confirmed the lower S-type lignin content in the *hub2* mutants and higher content in the *prn2-2* mutant compared with the WT (Fig. 3A). Both the *hub2-1 prn2-2* and *hub2-2 prn2-2* double mutants had S-type lignin content that was higher than in the WT (Fig. 3A), indicating that *prn2* is epistatic to *hub2*. The cross between *hub2-1* and the *PRN2* overexpression line *PRN2OE6* exhibited S-type lignin content similar to *PRN2OE6* (Fig. 3B). The result for the G-type lignin content was different, as both the *hub2* mutants and the *hub2 prn2* double mutants had a higher content of G-type lignin compared with the WT (Fig. 3A). However, *prn2* did not show consistent changes in the accumulation of G-type lignin (Figs 1A, 3A; Zhang *et al.*, 2020*a*), making it difficult to conclude on the epistatic relationship between HUB2 and PRN2 in G-lignin accumulation.

Next, we studied the promoter activity of *HUB2* in xylem tissues of mature stems and hypocotyls using pro*HUB2*::*GUS* transgenic plants. Consistent with a previous study (Cao *et al.*, 2008), the *HUB2* promoter was active in mature vascular tissues of the stem and the hypocotyl (Fig. 3C–E). The activity was, however, not evenly distributed; the strongest activity was found in the phloem and in close proximity to lignifying tissues of the primary and the secondary xylem (Fig. 3C–E). Promoter activity was present in cells expressing *PRN2*, namely the cells located next to the vessel elements (Zhang *et al.*, 2020*a*), but was never observed in the xylem vessel elements.

**Fig. 4.**
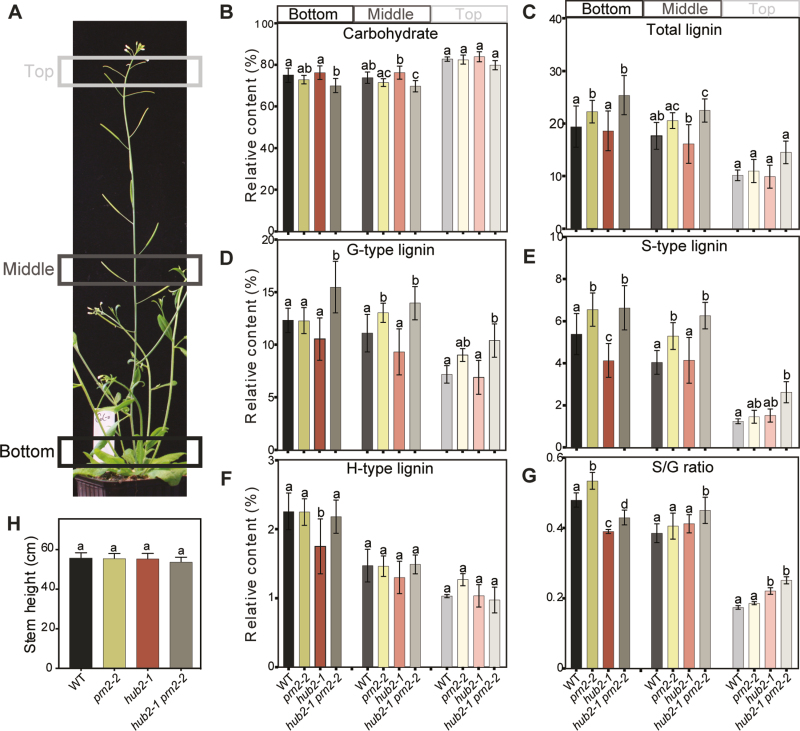
HUB2 influences the S-type lignin content of Arabidopsis stems. (A) Photograph illustrating the inflorescence stem fragments used for Py-GC/MS analysis. The bottom, middle, and top part of 55 cm long inflorescence stems of WT, *prn2-2*, *hub2-1*, and *hub2-1 prn2-2* were analysed. (B–G) Py-GC/MS analysis determining the relative amounts of carbohydrates (B), total lignin (C), G-type lignin (D), S-type lignin (E), and H-type lignin (F), and the S/G ratio (G) based on their respective MS peak area as a proportion of the cumulative area from all peaks (Gerber *et al.*, 2012). For each genotype, five biological replicates were analysed, each composed of a pool of three stem fragments. (H) The height of the inflorescence stems used for the analysis. Lines that do not share any letter are significantly different from each other according to post-ANOVA Fisher’s test (*n*≥3, *P*<0.05). Error bars indicate ±SD.

Taking into account the promoter activity of *HUB2* in not only hypocotyls but also stems, we next asked whether HUB2 and PRN2 have opposite effects on the S-lignin accumulation also in the stems. Total carbohydrate and lignin content as well as lignin composition were characterized in three different parts of the stem of *prn2-2*, *hub2-1*, and *hub2-1 prn2-2* by Py-GC/MS (Fig. 4). Similar to the hypocotyls, *prn2-2* and *hub2-1 prn2-2* displayed significantly increased S-type lignin content in the middle and bottom part of the stem. Likewise, *hub2-1* had significantly lower S-type lignin content in the bottom part of the stem, which was also reflected in the lower S/G-type lignin ratio observed in the mutant. The G-lignin and total lignin content followed the trends that were also apparent in the hypocotyl tissues of the different genotypes. These analyses therefore support the opposite function of HUB2 and PRN2 on S-lignin accumulation of the hypocotyl and, in particular, the mature parts of the stem.

### HUB2 is involved in the lignification of xylem cells

**Fig. 5.**
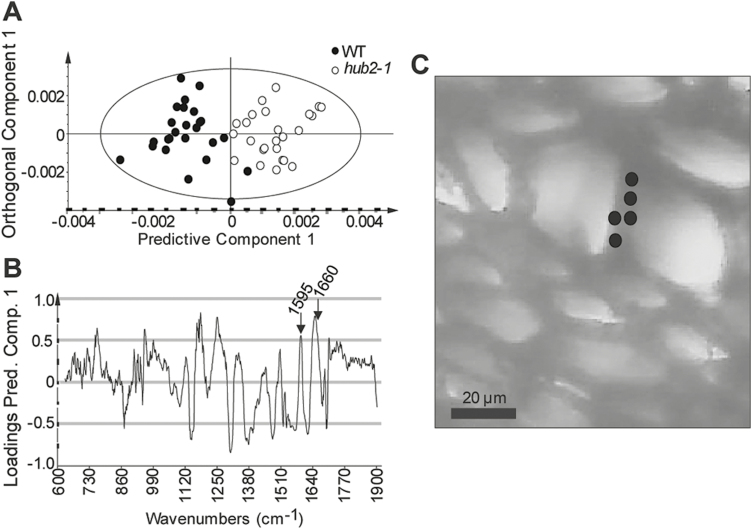
HUB2 alters the lignin properties of the xylem vessel secondary cell walls. (A, B) Raman microspectroscopic analysis of the secondary cell walls between two adjacent vessel elements in the secondary xylem of the hypocotyls in the *hub2-1* mutant and WT. Spectra were collected from five 8-week-old plants per genotype, with at least two images per plant and five spectra per image. OPLS-DA model with 1 + 2 (predictive+orthogonal) components, *n*=50, R2X(cum)=0.848, R2Y(cum)=0.746, Q2(cum)=0.69. The Q2(cum) value stands for the predictive ability of the model, with higher values (closer to the maximum 1) generally meaning better separation for the same data set. (A) Scores plot showing the separation between *hub2-1* (white symbols) and WT (black symbols) in the vessel–vessel walls. Each symbol represents one spectrum. (B) The corresponding correlation scaled loadings plot for the predictive component, showing factors separating the WT from *hub2-1* in vessel elements. Bands on the negative side of the plot have higher relative intensity in the spectra of WT plants, whereas bands on the positive side have higher relative intensity in the spectra of *hub2-1* plants. The bands corresponding to the aromatic -C=C- vibrations at 1595^–1^ and 1660 cm^–1^ (prominent bands associated with lignin) are marked by arrows. (C) White-light image of a representative transverse section analysed by Raman microspectroscopy. The circles show examples of locations extracted for spectra in cell walls between two adjacent vessel elements. The image is taken at ×50 magnification. The scale bar represents 20 µm.

To confirm the effect of HUB2 on the lignin landscape of xylem cell walls, individual xylem cell walls were chemotyped by Raman and FT-IR microspectroscopy in the *hub2* mutant hypocotyls (Fig. 5; Supplementary Fig. S2). Pairwise OPLS-DA (Bylesjö *et al.*, 2006) comparisons showed separation between the *hub2-1* mutant and the WT in the secondary cell walls between two vessels (the vessel–vessel walls, Fig. 5A). The corresponding loadings plot for the Raman microspectroscopic data revealed increased intensities for bands at 1595 cm^−1^ and 1660 cm^−1^ in *hub2-1* compared with the WT (Fig. 5B). Both bands can be attributed to lignin and suggest structural/compositional changes in addition to an increase in lignin content in *hub2-1*. In particular, the 1660 cm^−1^ band has been assigned to conjugated structures prevalent in coniferyl alcohols and coniferaldehydes (Gierlinger and Schwanninger, 2006). These results therefore support an increased proportion of G-type lignin in the walls of the vessel elements of *hub2-1*, which is in accordance with the overall increase in G-type and decrease in S-type lignin observed in the Py-GC/MS measurements of the bulk hypocotyl tissues of this mutant. We complemented the Raman microspectroscopy results with cell-specific FT-IR spectra in both vessel–vessel and fibre–fibre walls. OPLS-DA analysis of the FT-IR spectra showed separation between *hub2-1* and the WT in both cell types (Supplementary Fig. S2A, C). The loadings plot of the vessel–vessel walls indicated an overall increase in lignin content of *hub2-1* (increased abundance of the aromatic -C=C- bands around 1510 cm^−1^ and 1600 cm^−1^, Supplementary Fig. S2B) as well as lower proportions of cellulose (decreased abundance of –C-H band intensities around 1320–1360 cm^−1^) compared with the WT (Supplementary Fig. S2B). The corresponding loadings plot of the fibre–fibre walls revealed higher G-type/more cross-linked lignin (a proportionally higher increase of the 1510 cm^−1^ band, Supplementary Fig. S2D). The increased intensity of the –C=O vibration (around 1740 cm^−1^) and the decreased intensity of the –C-H vibrations in *hub2-1* FT-IR data (bands at 1320, 1360, and 1420 cm^−1^, Supplementary Fig. S2B, D) indicate further changes in the structure of the lignin–polysaccharide matrix. Taken together, the microspectroscopic results concur with the Py-GC/MS analysis done at the whole-tissue level. Both the Raman and the FT-IR analyses revealed proportionally increased G-lignin and thereby lower S/G ratios in *hub2*, supporting a role for HUB2 in stimulating accumulation of S-lignin at the expense of G-lignin.

### PRN2 does not seem to influence the H2Bub1 level of lignin biosynthetic genes

We hypothesized, based on the observed genetic interaction between HUB2 and PRN2 on S-type lignin accumulation, that the molecular function of PRN2 is related to the previously well-characterized role of HUB2 in mediating the H2Bub1 chromatin modifications in Arabidopsis (Fleury *et al.*, 2007; Cao *et al.*, 2008). We performed ChIP coupled to qPCR (ChIP-qPCR) to assess the H2BUb1 profile of lignin biosynthetic genes in hypocotyl tissues of mature *prn2-2* and WT plants. The analyses did not reveal any difference between *prn2-2* and the WT in the level of H2BUb1 of five selected lignin biosynthetic genes [*PAL* (phenylalanine ammonia-lyase), *C4H* (cinnamate 4-hydroxylase), *HCT* (*p*-hydroxycinnamoyl-CoA shikimate/quinate hydroxycinnamoyl transferase), *COMT* (caffeic acid 3-*O*-methyltransferase), and *F5H1* (ferulate 5-hydroxylase 1)] (Supplementary Fig. S3). Therefore, HUB2 and PRN2 may affect lignin content and composition through a mechanism other than controlling H2Bub1 levels in the chromatin regions of the selected lignin biosynthetic genes.

### PRN2 and HUB2 antagonistically regulate lignin-related genes

**Fig. 6.**
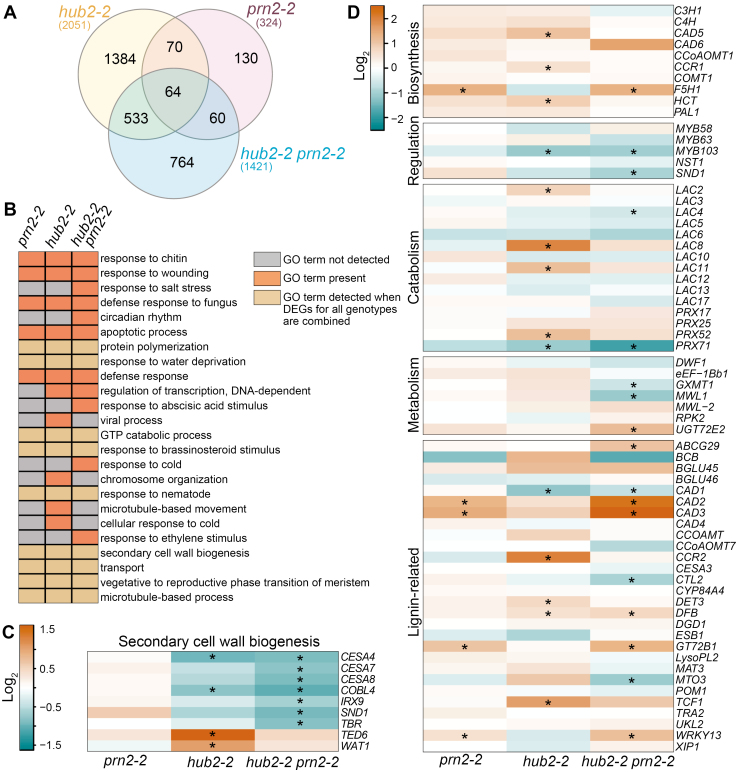
HUB2 and PRN2 regulate antagonistically the expression of *F5H1.* (A) Venn diagram showing the number of DEGs (differentially expressed genes) detected in an RNA-seq experiment in *hub2-2*, *prn2-2*, and *hub2-2 prn2-2*. The genes included in the analysis fulfil the criteria of |log_2_FC|>2 and a *P*_Adj_<0.05. For each genotype, three biological replicates were analysed, each consisting of a pool of five hypocotyls. The plants were grown under short-day (8/16 h) conditions for 7 weeks, followed by long-day (16/8 h) conditions for 4 weeks. (B) Enriched GO terms in all DEGs identified for each genotype. Enriched GO terms for DEGs in the individual mutants or DEGs combined for all mutants are indicated in orange and light brown, respectively. (C) Expression level of DEGs identified as part of the secondary cell wall biogenesis GO term (GO:0009834). (D) Expression level of all genes that are part of the lignin GO term (GO:0009809). Asterisks mark conditions in which gene expression is significantly different (following |log_2_FC|>2 and a *P*_Adj_<0.05) in the mutant compared with the wild type.

To elucidate whether the role of HUB2 and PRN2 in lignification is related to transcriptional regulation, RNA sequencing (RNA-seq) was performed on mature hypocotyls of *hub2-2*, *prn2-2*, and *hub2-2 prn2-2* plants. *hub2* and *hub2 prn2* mutants had 2051 and 1421 differentially expressed genes (DEGs), while *prn2* had only 324 DEGs (Fig. 6A). The differences in the abundance of DEGs were also reflected in the GO analysis of the DEGs (Fig. 6B). Several DEGs, mostly suppressed in both *hub2-2* and *hub2-2 prn2-2*, were observed within the secondary cell wall biogenesis GO category (Fig. 6C).

A specific focus was placed on lignin biosynthetic genes that would explain the opposite accumulation of S-type lignin in the *hub2* and *prn2* mutants. The most obvious candidate gene underlying the observed differences in S-lignin accumulation is *F5H1* which encodes the enzyme specific for S-type lignin biosynthesis. *F5H1* was indeed up-regulated in *prn2-2* and in *hub2-2 prn2-2,* but was down-regulated in *hub2-2* (Fig. 6D), in accordance with the S-lignin content of these lines (Figs 3A, 4E). The same criteria for gene expression were fulfilled by a few other lignin-related genes, such as *BCB* and *WRKY13* (Fig. 6D), but their function specifically in S-lignin biosynthesis is not known (Ji *et al.*, 2015; Li *et al.*, 2015). The expression of genes related to hemicellulose and cellulose, the two other major building blocks of secondary cell walls, was not affected (Supplementary Fig. S4). Taken together, the transcriptomic profiling supports the action of HUB2 and PRN2 on S-type lignin accumulation through transcriptional regulation of *F5H1*.

## Discussion

### HUB2 and PRN2 control S-lignin accumulation in opposite directions

Lignification of the cell walls is an irreversible process that needs to be tightly controlled during plant development and in response to various external stimuli in nature. A large number of transcription factors and other regulators (Taylor-Teeples *et al.*, 2015; reviewed by Kumar *et al.* 2016) ensure that lignin deposition takes place in an appropriate temporal and spatial manner. We demonstrated earlier that PRN2 suppressed accumulation of S-type lignin and was required to attain the correct, G-enriched lignin composition of the vessel elements in Arabidopsis stems and hypocotyls in a non-cell-autonomous manner (Zhang *et al.*, 2020*a*). The current study demonstrates the role of HUB2, a protein interactor of PRN2, in xylem cell wall lignification. HUB2 stimulates S-type lignin accumulation in both hypocotyls and stems (Figs 1, 3, 4). HUB2 therefore has an opposite function to PRN2, and it is likely on the basis of the protein interaction and genetic analyses that PRN2 and HUB2 function together in control of S-type lignin accumulation and that PRN2 acts downstream of HUB2 in this process (Fig. 3A, B).

The non-cell-autonomous function of PRN2 in lignification of the vessel elements was revealed by the fact that PRN2 is not itself localized in vessel elements, but rather in cells next to the vessels (Zhang *et al.*, 2020*a*). The *HUB2* promoter was active in many different kinds of cells, including the PRN2-expressing cells, but no activity was observed in the vessel elements (Fig. 3). High-resolution gene expression analysis in the root does not show expression of *HUB2* in vessel elements either (Brady *et al.*, 2007). The expression pattern therefore supports a role for HUB2 in promoting S-type lignin accumulation in most xylem cells but not vessel elements. The stimulatory role of HUB2 on S-type lignin accumulation is counteracted by PRN2 in cells surrounding the vessel elements, to prevent accumulation of S-type lignin that is less resistant than G-type lignin to the mechanical constraints generated by water transport in the vessel elements.

### HUB2 and PRN2 diverge on the transcriptional regulation of *F5H1* expression

It was proposed earlier that PRN2 suppresses S-type lignin biosynthesis by transcriptional suppression of the biosynthetic genes and some of the secondary cell wall-related transcription factors (Zhang *et al.*, 2020*a*). This prompted us to investigate whether HUB2 is also involved in transcriptional regulation of lignification and to scrutinize the hierarchy of the pathway in which HUB2 and PRN2 operate. The *hub2* mutant displayed alterations in the expression of a wide array of genes, including those related to regulation or biosynthesis of lignin. However, only a few lignin-related genes were altered in the directions expected for the suppressive role of PRN2 and the stimulatory role of HUB2 in S-type lignin accumulation. Strikingly, one of these genes was *F5H1* which is the key gene in S-type lignin biosynthesis. Even though the details of the HUB2–PRN2 interaction in S-type lignin accumulation remain to be further elucidated, *F5H1* clearly seems to be involved in this process.

The fact that HUB2 facilitates monoubiquitination of histone 2B (H2BUb1) in chromatin (Fleury *et al.*, 2007) prompted us to elucidate whether HUB2-mediated H2BUb1 chromatin modification underlies changes in the expression of the lignin biosynthetic genes, including *F5H1*. We could not, however, detect changes in H2BUb1 levels of *F5H1* in the *hub2* mutant (Supplementary Fig. S3). It therefore seems that HUB2 function in S-type lignin biosynthesis is not related to H2BUb1 of *F5H1* chromatin even though we cannot exclude the possibility that HUB2-mediated H2BUb1 of lignin-related genes could have spatial or temporal dynamics that have not been captured in this study. Besides their role in chromatin modifications, E3 ubiquitin ligases, such as HUB2, have been implicated in ubiquitin-dependent proteolysis. A recent study in cotton proposed that the functional homologue of AtHUB2, GhHUB2, is involved in protein degradation via the ubiquitin–26S proteasome pathway (Feng *et al.*, 2018). We cannot therefore rule out that the function of HUB2 in lignification is somehow related to proteolysis.

In conclusion, our study describes HUB2 as a protein interactor of PRN2, and the involvement of HUB2 in lignification. We show that HUB2 and PRN2 regulate S-type lignin accumulation in opposite directions by controlling the expression of *F5H1*.

## Supplementary data

Supplementary data are available at *JXB* online.

**Fig. S1.** HUB2 does not affect secondary xylem anatomy and cell morphology in Arabidopsis.

**Fig. S2.** FT-IR analysis of xylem vessel elements and fibres in Arabidopsis hypocotyl.

**Fig. S3.** The abundance of H2BUb1 chromatin marks of five lignin biosynthetic genes in stem tissues.

**Fig. S4.** Expression profile of hemicellulose- and cellulose-related genes.

**Table S1.** All mutants used in this study.

**Table S2.** All primer sequences used in this study.

eraa264_suppl_Supplementary_Material

## Data Availability

The following datasets are available at the Dryad Data Repository: https://dx.doi.org/10.5061/dryad.mgqnk98wf (Zhang et al., 2020*b*). Dataset 1. Yeast two-hybrid screen using the full-length Arabidopsis PRN2 as a bait. Dataset 2. Comparative transcriptome analysis.
