# Supplementary material for: The chromatin-modifying protein HUB2 is involved in the regulation of lignin composition in xylem vessels
Source: J Exp Bot. 2020 Jun 1;71(18):5484–94. doi: 10.1093/jxb/eraa264 (PMC7501814; doi:10.1093/jxb/eraa264)
Supplement: eraa264_suppl_Supplementary_Material [file eraa264_suppl_supplementary_material.pdf]

The chromatin-modifying protein HUB2 is involved in the regulation of lignin composition in xylem vessels

Bo Zhang, Bernadette Sztojka, Carolin Seyfferth, Sacha Escamez, Pál Miskolczi, Maxime Chantreau, László Bakó, Nicolas Delhomme, András Gorzsás, Rishikesh P. Bhalerao, and Hannele Tuominen

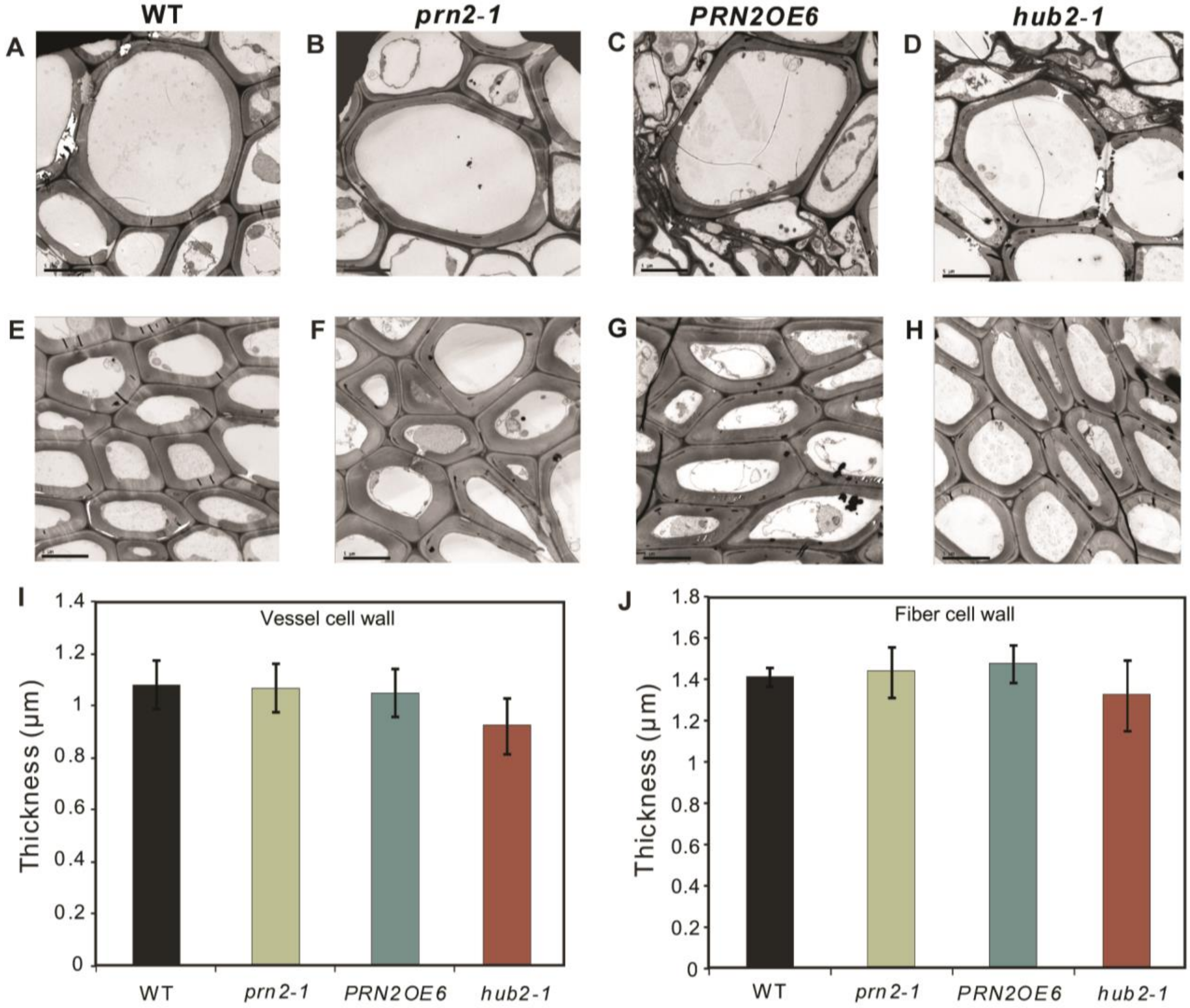

Supplementary Figure 1. Electron microscopy analysis of the secondary xylem cell morphology
Electron microscopy analyses of representative vessel elements (A-D) and fibers (E-H) in secondary xylem tissues of the hypocotyls in WT (A, E), *prn2-1* (B, F), *PRN2OE6* (C, G) and *hub2-1* (D, H). The transverse sections were derived from the hypocotyls of 8-week-old, short-day-grown plants.
Bar = 1 µm.
(I, J) Cell wall thickness of vessel elements (I) and fibers (J) in the secondary xylem tissues of the hypocotyls. The thickness (from lumen edge to middle lamella) was measured by ImageJ, at more than 4 positions of each cell. Eight to ten cells were measured for each biological replicate and three biological replicates were analyzed for each genotype. Error bars indicate ±SD. No statistical differences were detected between the different genotypes.

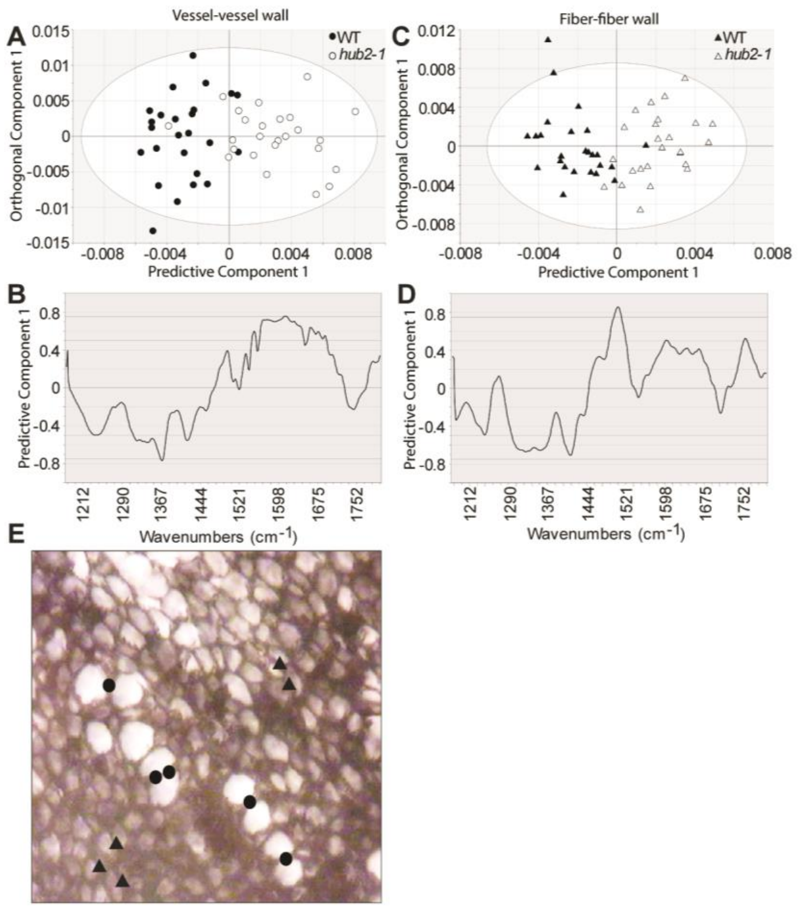

Supplementary Figure 2. FT-IR analysis of xylem vessel elements and fibers in the Arabidopsis hypocotyl.

(A-D) FT-IR microspectroscopic analysis of vessel elements (A,B) and fibres (C,D) in the secondary xylem of the hypocotyls in the *hub2-1* mutant and WT. Spectra were collected from five 8-week-old plants per genotype, with a minimum of five spectra per plant and cell type. OPLS-DA models used 1+2 (predictive + orthogonal) components, with the following details: vessel elements: N = 50, R2X(cum) = 0.729, R2Y(cum) = 0.621, Q2(cum) = 0.520; fiber cells: N = 50, R2X(cum) = 0.651, R2Y(cum) = 0.689, Q2(cum) = 0.563. The Q2(cum) value stands for the predictive ability of the model, with higher values (closer to the maximum 1) meaning better separation for the same dataset. (A, C) OPLS-DA scores plots showing the separation between *hub2-1* (white symbols) and WT (black symbols) in vessel–vessel walls (A) and cell walls between two fiber cells (C). Each symbol represents one spectrum. (B, D) The corresponding correlation-scaled loading plots for the predictive components, showing factors separating WT from *hub2-1* in vessel elements (B) and fiber cells (D). Bands on the negative side of the plots have higher relative intensity in the spectra of WT, while bands on the positive side have higher relative intensity in the spectra of *hub2-1*.

(E) White-light image of a transverse section showing example positions of the extracted representative FT-IR spectra for cell walls between two vessel elements (circles), and between two xylem fibres (triangles). The image was taken at the fixed ×15 magnification of the Cassegrain objective of the FT-IR microscope.

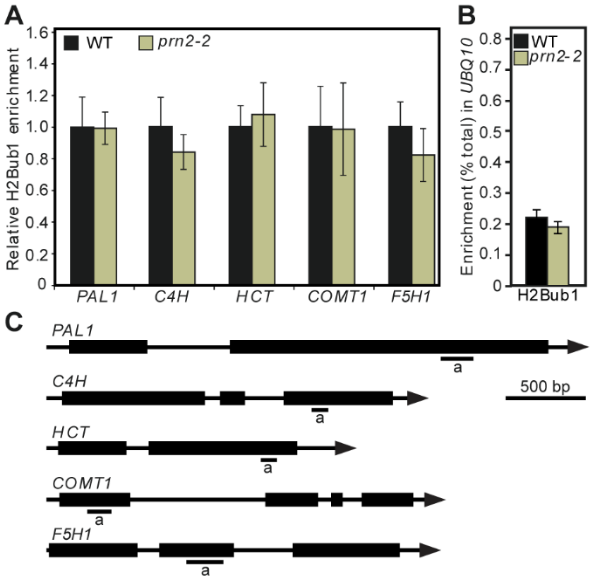

Supplementary Figure 3. The abundance of H2BUb1 chromatin marks of lignin-biosynthetic genes in stem tissues.

(A) Enrichment of H2BUb1 marks relative to the WT in the gene body of selected lignin-biosynthetic genes. ChIP was followed by qPCR in *prn2-2* and WT plants. The data was normalized to H2Bub1 levels of UBQ10 chromatin (see panel B). The data was further normalized to the WT level.

(B) H2Bub1 levels at the Arabidopsis UBQ10 (AT4G05320) locus in WT and *prn2-2* stems.

For each genotype, three biological replicates were analyzed, each composed of a pool of two cm bottom parts of 50-cm-tall main stems from fifteen plants. Error bars indicate ±SD. All ChIP data were normalized to the input chromatin.

(C) Schematics representing the genomic structure of the analyzed genes. Black rectangles represent exons, black lines introns and untranslated regions, the arrows indicate the direction of transcription. The genomic fragments targeted by ChIP-qPCR are marked with bars labeled with “a”.

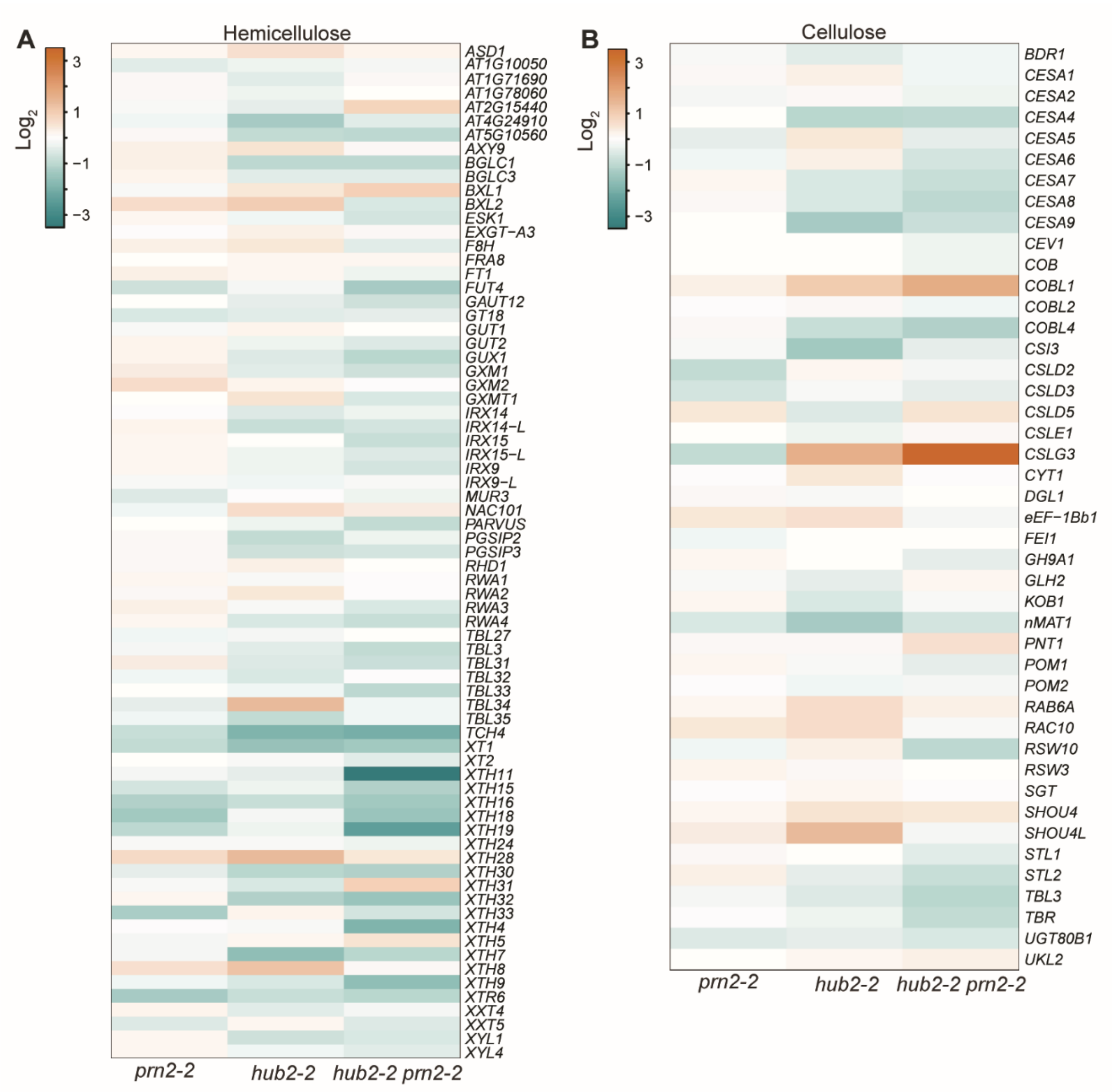

Supplementary Figure 4. Expression profile of hemicellulose- and cellulose-related genes. The expression level of all genes belonging to the hemicellulose metabolic (A) (GO:0010410) and cellulose biosynthetic process (B) (GO:0030244) GO terms analyzed in *prn2-2*, *hub2-2* and *hub2-2 prn2-2*.

Supplementary Table 1. All mutants used in this study		
**T-DNA line**	**Gene ID**	**Gene Name**
rd21-1 (SALK_090550C)	AT1G47128	cysteine proteinase (RD21A) / thiolprotease
GK-401H08	AT1G47128	cysteine proteinase (RD21A) / thiolprotease
rd21-1 (SALK_065256C)	AT1G47128	cysteine proteinase (RD21A) / thiolprotease
SALK_049838C	AT1G63770	peptidase M1 family protein
SALK_047811C	AT1G63770	peptidase M1 family protein
SALK_062487	AT1G63770	peptidase M1 family protein
SALK_111997C	AT1G20850	XCP2
SALK_057921C	AT1G20850	XCP2
xcp2 (SALK_010938)	AT1G20850	XCP2
SALK_079305C	AT2G25740	ATP-dependent protease La (LON) domain-containing protein
hub2-1 (GK634H04)	AT1G55250	HUB2
hub2-2 (SALK_071289)	AT1G55250	HUB2
itn-t (GK-366G12.01)	AT3G12360	ITN1
SALK_043552	AT4G24620	PGI1
SALK_015973	AT4G24620	PGI1
SALK_136720C	AT1G56000	amineoxidase-related
SALK_091124C	AT3G07340	basic helix-loop-helix (bHLH) family protein
SALK_043364	AT1G60070	clathrin binding (AT1G60070)
SALK_026138	AT5G07400	forkhead-associated domain-containing protein / FHA domain-containing protein
SALK_080172C	AT1G42430	hypothetical protein AT1G42430
SAIL_245_A02	AT5G22450	hypothetical protein AT5G22450
SALK_020799C	AT2G05920	subtilase familyprotein
GK-168E04.01	AT2G05920	subtilase familyprotein
SALK_137002C	AT5G07740	formin homology 2 (FH2) domain-containing protein,
SALK_031363C	AT5G23110	zinc finger (C3HC4-type RING finger) familyprotein
SALK_069537C	AT5G23110	zinc finger (C3HC4-type RING finger) familyprotein
prn2-1 (SM_3.15394)	AT2G43120	PIRIN2
prn2-2 (SALK_079571)	AT2G43120	PIRIN2
ccr1-3 (SALK_123689)	AT1G15950	CINNAMOYL COA REDUCTASE 1
c4h-3 (ref3.3)	AT2G30490	CINNAMATE 4-HYDROXYLASE

Supplementary Table 2. All primer sequences used in this study

**Sequence**	**Primer Name**	**Purpose**
TGTGACCTTTGAAGTCTTGGTG	SM_3_15394-L	*prn2-1* T-DNA line genotyping
CTCGTTTTTCTGTCATTTGACTG	SM_3_15394-R	*prn2-1* T-DNA line genotyping
CCGAATCAACAACAATGAAAAC	SALK_079571.50.85.x-L	*prn2-2* T-DNA line genotyping
TGTGACCTTTGAAGTCTTGGTG	SALK_079571.50.85.x-R	*prn2-2* T-DNA line genotyping
TTGCTGCTTTTAGCTCGAGAG	GK-168E04-LP	AT2G05920 T-DNA line genotyping
TTGGGATGCTTTCACCTACAC	GK-168E04-RP	AT2G05920 T-DNA line genotyping
ACCACACTATCAGGGTGATGC	GK-366G12-LP	AT3G12360 T-DNA line genotyping
CGAACCACCCATATGTTTCAC	GK-366G12-RP	AT3G12360 T-DNA line genotyping
TCCATCAACACCCTTGTAAGG	GABI_401H08-LP	AT1G47128 T-DNA line genotyping
CTGAAGAAGAAATGGGGTTCC	GK-401H08-RP	AT1G47128 T-DNA line genotyping
ATTGCATATGCTAACGGATGC	GK-634H04-LP	AT1G55250 (*hub2-1*) T-DNA line genotyping
GGCTTTCCGAAAAGGTATCAC	GK-634H04-RP	AT1G55250 (*hub2-1*) T-DNA line genotyping
CATGGTACCACATCCAAGGTC	SALK_071289.51.25-LP	AT1G55250 (*hub2-2*) T-DNA line genotyping
CCTCTTTAGGCCGATCAAAAC	SALK_071289.51.25-RP	AT1G55250 (*hub2-2*) T-DNA line genotyping
CGATTCTCACTGTGGTTCATG	SAIL_245_A02-LP	AT5G22450 T-DNA line genotyping
CTAGCAACTGTCCGCCTAATG	SAIL_245_A02-RP	AT5G22450 T-DNA line genotyping
AAAGGGAAAAGCTACTGGCTC	SALK_010938.56.00-LP	AT1G20850 T-DNA line genotyping
GGTTTCCCAGTGTTCCTCTTC	SALK_010938.56.00-RP	AT1G20850 T-DNA line genotyping
CCAGGAAGTGACACCAACATC	SALK_015973.55.00-LP	AT4G24620 T-DNA line genotyping
TCGAATTTCAAAAGTGGCAAG	SALK_015973.55.00-RP	AT4G24620 T-DNA line genotyping
CACAATCGCAACAATGTGATC	SALK_020799.55.00-LP	AT2G05920 T-DNA line genotyping
TGTTGCTCCTTGGGTTATGAC	SALK_020799.55.00-RP	AT2G05920 T-DNA line genotyping
GAGGGAGGATGAGGATGACTC	SALK_026138.51.75-LP	AT5G07400 T-DNA line genotyping
TACAGCATTTTGACTCCCAGG	SALK_026138.51.75-RP	AT5G07400 T-DNA line genotyping
AGCAAGAGGTAGCAGGGGTAG	SALK_031363.18.65-LP	AT5G23110 T-DNA line genotyping
TACTGCATCGTGTTTGCAAAG	SALK_031363.18.65-RP	AT5G23110 T-DNA line genotyping
TTTCTGATGATCCTTATCGCG	SALK_043364.56.00-LP	AT1G60070 T-DNA line genotyping
TCATGAATCCATTCTCTTCCG	SALK_043364.56.00-RP	AT1G60070 T-DNA line genotyping
AAAGTAGGCTTAGGTGCGAGC	SALK_043552.29.80-LP	AT4G24620 T-DNA line genotyping
AATCGACATCCACGCATCTAG	SALK_043552.29.80-RP	AT4G24620 T-DNA line genotyping
CATGCCAACTTCTCTGTCCTC	SALK_047811.56.00-LP	AT1G63770 T-DNA line genotyping

CCTGGAGTTGGAGGTATCTCC	SALK_047811.56.00-RP	AT1G63770 T-DNA line genotyping
GTTCTGGCAAGAAAGCTGATG	SALK_049838.54.75-LP	AT1G63770 T-DNA line genotyping
TGTAACCTGATCCATCCTTGG	SALK_049838.54.75-RP	AT1G63770 T-DNA line genotyping
GACACTGAGAGGCTGATGAGC	SALK_057921.45.15-LP	AT1G20850 T-DNA line genotyping
AGCGACCTCTATCGAGTCTCC	SALK_057921.45.15-RP	AT1G20850 T-DNA line genotyping
ACATCCTCATCCCACTTCATG	SALK_062487.17.35-LP	AT1G63770 T-DNA line genotyping
TTGCATGAGCTGTTTGCATAG	SALK_062487.17.35-RP	AT1G63770 T-DNA line genotyping
CTGAAGAAGAAATGGGGTTCC	SALK_065256.45.05-LP	AT1G47128 T-DNA line genotyping
GTTTATTCCCTCCACTGCTCC	SALK_065256.45.05-RP	AT1G47128 T-DNA line genotyping
TGGAGATTCGATTGGTCAAAG	SALK_069537.54.00-LP	AT5G23110 T-DNA line genotyping
TGTAACACAGTGACCCCAAGG	SALK_069537.54.00-RP	AT5G23110 T-DNA line genotyping
CGAGTAAAATTCCCGTGTCTG	SALK_079305.41.45-LP	AT2G25740 T-DNA line genotyping
AAAAATCTAATGCTCAGCGGC	SALK_079305.41.45-RP	AT2G25740 T-DNA line genotyping
TTCACCAACTTTCGAATTTGG	SALK_080172.48.00-LP	AT1G42430 T-DNA line genotyping
TTCCTCTGACTTCTCAGAGCG	SALK_080172.48.00-RP	AT1G42430 T-DNA line genotyping
GAAAGCAGTTGCTCATCAACC	SALK_090550.52.85-RP	AT1G47128 T-DNA line genotyping
ATACACGAAACCCAACAGCTG	SALK_090550.52.85-LP	AT1G47128 T-DNA line genotyping
CACCCAATAACGTTTCTGCTG	SALK_091124.40.35-LP	AT3G07340 T-DNA line genotyping
AAAATGCCACGTGTATCAAGC	SALK_091124.40.35-RP	AT3G07340 T-DNA line genotyping
TCACCGTAATCTCATGTTTTGTG	SALK_111997.34.15-LP	AT1G20850 T-DNA line genotyping
CCACAGCTCCTTTCTTTCTCC	SALK_111997.34.15-RP	AT1G20850 T-DNA line genotyping
TTCCTCACTCTTGTACCCACG	SALK_136720.45.10-LP	AT1G56000 T-DNA line genotyping
TTTGGGGTAAGATTGCTTCAG	SALK_136720.45.10-RP	AT1G56000 T-DNA line genotyping
TTCCCTGAAGCCATTACACTG	SALK_137002.14.85-LP	AT5G07740 T-DNA line genotyping
GTAGCTCCATCTCCTCCTTGG	SALK_137002.14.85-RP	AT5G07740 T-DNA line genotyping
TACGAATAAGAGCGTCCATTTTAGAGTGA	spm	for SM T-DNA lines genotyping
ATTTTGCCGATTTCGGAAC	LBb1.3	for SALK T-DNA lines genotyping
GCCTTTTCAGAAATGGATAAATAGCCTTGCTTCC	LB1	for SAIL T-DNA lines genotyping
CCA GGATCC ATGAGAGCTGCAATAAACAGAGC	AtPRN2-3F	For construction of pRT104(3xHA/3xMyc)PRN2
CCA CAATTG TCATTGTGACCTCCAATA	AtPRN2-3R	For construction of pRT104(3xHA/3xMyc)PRN2
CCA GGATCC ATGGAGAATCAGGAATCGGACGAGCC	AtHUB2.1-1F	For construction of pRT104(3xHA/3xMyc)HUB2
CCC ATCGAT TTACATTTTGACAAGCCGGACG	AtHUB2.1-1R	For construction of pRT104(3xHA/3xMyc)HUB2
TCACCATTGACTCCAGTAGTAAGA	AtPAL1-chip-F	For ChIP-qPCR analysis of PAL1 (At2g37040)

CCAAGATGTCAACTCTTTGGGAC	AtPAL1-chip-R	For ChIP-qPCR analysis of PAL1 (At2g37040)
GGCACGAGGAGAGGAATCGCC	AtC4H-chip-F	For ChIP-qPCR analysis of C4H (At2g30490)
ACCGGGTGTGCAAGTCACCG	AtC4H-chip-R	For ChIP-qPCR analysis of C4H (At2g30490)
CTGTGGATGATGGGGATGTTGTC	AtF5H1-chip-F	For ChIP-qPCR analysis of F5H1 (At4g36220)
TGCGAGAAGGGACAAGACGAG	AtF5H1-chip-R	For ChIP-qPCR analysis of F5H1 (At4g36220)
GCCAGTGCTTCCGTTCTTCCGATG	AtCOMT-chip-F	For ChIP-qPCR analysis of COMT (At5g54160)
CGGTCGAGCATGACCGGAGC	AtCOMT-chip-R	For ChIP-qPCR analysis of COMT (At5g54160)
CCCTTGTCCGCGGTGCACAT	AtHCT-chip-F	For ChIP-qPCR analysis of HCT (At5g48930)
GGACGACCCCAACCAAAGTCTGC	AtHCT-chip-R	For ChIP-qPCR analysis of HCT (At5g48930)
GGGCCTTGTATAATCCCTGATGAATAAGTG	AtUBQ10-F	housekeeping gene (AT4G05320) for ChIP-qPCR
AAAGAGATAACAGGAACGGAAACATAGT	AtUBQ10-R	housekeeping gene (AT4G05320) for ChIP-qPCR
